# Post-traumatic stress disorder among people exposed to the Ventotene street disaster in Rome

**DOI:** 10.1186/1745-0179-4-5

**Published:** 2008-03-05

**Authors:** Michele Raja, Antonio Onofri, Antonella Azzoni, Bruno Borzellino, Nicoletta Melchiorre

**Affiliations:** 1 Università di Roma, La Sapienza, Rome, Italy; 2 Servizio Psichiatrico di Diagnosi e Cura, Ospedale Santo Spirito, Rome, Italy

## Abstract

**Objective:**

To test five hypotheses on Post-traumatic stress disorder (PTSD): 1) Is PTSD the most prevalent disorder after trauma? 2) Is the proximity to the disaster related to the risk of PTSD? 3) Is PTSD associated with child mourning or separation, previous stress, or familiarity for psychiatric disorders? 4) Does the exposition to trauma increase substance abuse or somatization? 5) Can episodic trauma cause long-lasting psychiatric morbidity?

**Methods:**

Clinical assessment of subjects exposed to an explosion in a building caused by a gas-leak. Best estimate clinical diagnoses were made according to DSM-IV-TR criteria. The Zung Depression Rating Scale, the Zung Anxiety Rating Scale, and the Clinician Administered Post Traumatic Stress Disorder Scale were used in the clinical assessment. Statistical analysis was performed by means of t-test with Bonferroni's correction on continuous variables and χ^2 ^or Fisher test on categorical variables.

**Results:**

PTSD was the most prevalent disorder after trauma, diagnosed in 32 (36.8%) subjects. The subjects who had not seen dead or injured people were more likely to receive no psychiatric diagnosis. Civil status, parenthood, death of relatives in the disaster, personal injuries, history of child mourning or separation, of previous stress, as well as familiarity for any psychiatric disorder or substance use disorder were not related with the rate of ascertained psychiatric diagnoses. Nearly two years after trauma, most of patients who had suffered PTSD still met PTSD criteria.

**Conclusion:**

The 1^st ^and the 5^th ^hypotheses were corroborated, the 3^rd ^and the 4^th ^hypotheses were not confirmed. The 2^nd ^hypothesis was partially confirmed.

## Introduction

Our understanding of post-traumatic stress disorder (PTSD) relies predominantly on studies of war veterans and disaster victims. Estimates of PTSD prevalence tend to vary according to the diagnostic criteria used to define the disorder, assessment procedures, sample characteristics, and the definition of traumatic events. Possible reasons for the observed difference in lifetime prevalence of PTSD between the sexes (a female-to-male lifetime prevalence ratio of 2:1 is typically reported) and factors thought to be associated with an increased risk for the disorder after exposure to trauma are unclear [1]. PTSD has been a controversial construct because of the complex factors that have been hypothesized to influence its onset and prevalence, such as compensation and withdrawal from combat duty.

To complicate the picture, a considerable number of partial and subthreshold syndromes exists [2].

The lifetime prevalence of PTSD in the United States is 8 to 9%, and approximately 25 to 30% of victims of trauma develop PTSD [3]. PTSD may affect survivors of accidents and illnesses, in addition to violence victims and combat veterans. It is unlikely to find out consistent results studying populations exposed to heterogeneous stresses. Furthermore, medico-legal issues may introduce bias.

This paper attempts to draw together some of the current questions related to the methodology of exploring the psychiatric aspects of human response to civil disasters.

In November 2001, a gas-leak caused an explosion in a building in *Ventotene *street in Rome damaging several buildings, killing eight persons (four members of the Fire Brigade), and injuring decades of people. We had the opportunity to visit and assess psychiatric conditions of all the survivors living in the building where the explosion occurred. The evaluation was conducted in interview sessions 20 months after the disaster to assess the impact of the disaster on survivors' anxiety, depression, and post-traumatic stress, and to examine post-disaster rates of disorders and symptoms.

The aim of the present study was to corroborate five hypotheses suggested by the available evidence on PTSD. First, PTSD is the most prevalent psychiatric disorder after trauma; second, there is a positive relation between the proximity to the disaster and the risk of suffering from PTSD; third, PTSD is associated with history of child mourning or separation, previous stress, or familiarity for psychiatric disorder; fourth, exposition to trauma increases substance abuse disorder, or somatization; fifth, episodic trauma may cause long-lasting psychiatric morbidity.

## Subjects and Methods

Subjects (N = 101) received a diagnostic and clinical assessment at 20 months post-disaster. Assessments were made by four senior psychiatrists with at least 20 years clinical experience. Diagnoses were made according to DSM-IV-TR criteria. Information was gathered retrospectively from patients' reports, clinical notes and legal reports. The following data were ascertained for each patient: sex, age, years of education, civil status, parenthood, diagnosis, past and current psychopharmacological or psychotherapeutic treatment. Furthermore, we ascertained whether a subject had relatives with psychiatric disorder or substance abuse, had been present at the moment of the explosion, had had relatives dead in the disaster, had suffered personal injures or disruptive consequences on work, and had seen people injured or dead in the disaster.

The clinical conditions were assessed by a clinical interview. Furthermore, current psychopathology was assessed using the Zung Depression Rating Scale, the Zung Anxiety Rating Scale, and the Clinician Administered Post Traumatic Stress Disorder Scale (CAPS). The CAPS [4] is a clinical interview dedicated to PTSD that quantifies PTSD symptoms' frequency and intensity, and therefore yields a continuous measure of symptom severity as well a dichotomous diagnosis of PTSD. The duration of the time frame for assessment was the last month. PTSD identifies the most severe trauma victims, who are markedly distinguishable from victims with subthreshold PTSD [5]. We conducted statistical analysis by means of t-test with Bonferroni's correction on continuous variables and χ^2 ^or Fisher test on categorical variables. P < 0.05 was considered to be statistically significant.

## Results

Men were 47 and women 54. Seven men and seven women, who were less than 18-year old, did not receive a complete psychiatric assessment and were excluded from analysis. Forty men and 47 women entered the study. Subjects' mean age was 43.9 (± 16.7) years. The socio-demographic and clinical features of the subjects are shown in Table 1. Life-time PTSD was diagnosed in 43 (49.4%). At the time of assessment, 32 (36.8%) subjects still suffered from PTSD. It was severe or very severe in 15 and moderate in 17 subjects. Six (6.9%) subjects received a diagnosis of Generalized Anxiety Disorder (GAD), and 49 (56.3%) subjects received no psychiatric diagnosis. At the time of assessment, current CAPS score was significantly higher in women (40.0 ± 25.8) than in men (27.1 ± 19.9) [t = 2.228; df = 84; p = 0.025], as well as Zung Depression Scale score (women: 50.1 ± 1.7; men: 43.1 ± 11.2; t = 2.721; df = 85; p = 0.008), and Zung Anxiety Scale score (women: 50.9 ± 12.7; men: 40.8 ± 11.7; t = 3.835; df = 85; p = 0.000). However, the categorical diagnosis of PTSD was not significantly more frequent among women (21/47, 44.7%) than men (11/40, 27.5%) (p = 0.152). For purposes of data analysis, we considered three groups: subjects with no current psychiatric diagnosis, subjects with current GAD, and subjects with current PTSD. The CAPS score was higher in patients with current PTSD (58.2 ± 18.0) than in subjects with no psychiatric diagnosis (18.6 ± 13.0), and GAD (26.0 ± 10.1)) [variance analysis: F = 67.05; p = 0.000; Bonferroni test). The Zung Depression Scale score was higher in patients with current PTSD (54.2 ± 10.7) than in patients with GAD (51.2 ± 13.3) and in subjects with no psychiatric diagnosis (41.6 ± 12.1) [variance analysis: F = 13.23; p = 0.000; Bonferroni test). The Zung Anxiety Scale score was higher in patients with current PTSD (53.8 ± 10.3) and in patients with GAD (56.8 ± 8.7) than in subjects with no psychiatric diagnosis (40.1 ± 12.1) [variance analysis: F = 17.21; p = 0.000; Bonferroni test). The presence at the moment of the explosion was not correlated with any of the three diagnostic groups. The subjects who had not seen dead or injured people (N = 58) were more likely to receive no psychiatric diagnosis than a diagnosis of PTSD or a diagnosis of GAD (p = 0.002). Civil status, parenthood, death of relatives in the disaster, personal injuries, history of child mourning or separation, of previous stress, as well as familiarity for any psychiatric disorder or substance use disorder were not related with the rate of ascertained psychiatric diagnoses. Suicidal behavior in relatives was more frequent among subjects with PTSD than among subjects with GAD or no psychiatric diagnosis (p = 0.010). Patients with PTSD were more likely taking psychoactive drugs than subjects with no psychiatric diagnosis (p = 0.003).

**Table 1:** Socio-demographic and clinical variables in the subjects with PTSD, GAD, and no psychiatric diagnosis

	PTSD	GAD	No psychiatric diagnosis	Χ^2^	df	p
Gender (men/women)	11/21	2/4	27/22	3.763	2	0.152
Civil status (single/married/separated or widow-wer)	8/18/6	2/4/0	16/30/3	4.187	4	0.381
Education level (low/medium/high)	8/14/10	1/1/4	9/16/24	3.954	4	0.412
Parenthood	20/12	3/3	27/19	0.358	2	0.837
Presence at the moment of the explosion (y/n)	21/11	3/3	24/25	2.238	2	0.327
Sight of dead or injured people	16/15	4/2	8/41	14.184	2	0.000*
dead relatives (y/n)	1/31	0/6	5/44	1.988	2	0.370
personal injures (y/n)	4/28	0/6	3/46	1.628	2	0.443
disruptive consequences on work (y/n)	19/13	3/3	15/34	6.699	2	0.035*
psychiatric disorders in relatives (y/n)	5/27	0/6	4/45	1.906	2	0.386
substance abuse disorders in relatives (y/n)	4/28	0/6	2/47	2.614	2	0.271
Suicidal behavior in relatives (y/n)	5/27	0/6	0/49	9.118	2	0.010*
previous severe trauma (y/n)	4/28	0/6	2/47	2.614	2	0.271
mournings in childhood (y/n)	12/20	1/5	9/40	4.004	2	0.135
separation in childhood (y/n)	5/27	0/6	2/47	4.050	2	0.132
previous psychiatric disorder (y/n)	6/26	0/6	5/43	2.141	2	0.343
Current psychopharmacological treatment (y/n)	15/17	3/3	7/42	8.810	2	0.003*
Current psychotherapy (y/n) (y/n)	6/26	0/6	3/45	3.956	2	0.138

PTSD = Post-traumatic stress disorder; GAD = Generalized anxiety disorder

* = statistically significant

## Discussion

### Limitations

There are several limitations in the study that need to be acknowledged.

First, PTSD is being used and abused in compensation claims. The study was conducted by psychiatrists of a public hospital with no conflict of interest. However, at the time of the study, a legal controversy was in course between subjects exposed to the disaster and the gas Company. Therefore, some subjects might have emphasized or simulate symptoms in order to obtain legal compensation. It has been reported that disability compensation incentives influence the way some veterans report their symptoms when they are being evaluated for PTSD [6]. While it is not possible to rule out this possibility definitively, the consistency of most results with previous studies suggests that they are reliable and that the medico-legal context played a minor role (if any) in influencing the results. The authors did not receive the impression that subjects tended to manipulate or malinger.

Second, life-time diagnoses were retrospective and structured interviews were not used in the assessment of psychiatric disorders different from PTSD. This may account for the relatively low prevalence of psychiatric disorders observed in the studied population. However, some reliable key questions (sistematically asked to all patients) – such as personal or relatives' previous psychiatric hospitalization, attempted suicide, psychopharmacological treatment, psychotherapy, alcohol or drug abuse – suggest that clinically significant psychiatric disorders were correctly assessed in the sample. Furthermore, it should be noted that a significant number of subjects with no current psychiatric diagnosis made at the time of assessment was receiving pharmacotherapy prescribed by treating physicians. This discrepancy suggests that patients did not exaggerate their symptoms at the time of assessment and that strict diagnostic criteria were used by authors.

Third, the number of examined subjects is small. Some differences may have resulted not statistically significant because of the small size of the examined groups. For example, women were more likely to present PTSD than men, although the difference failed to reach statistical significance probably for the little sample size.

Fourth, subjects who were less than 18-year old were excluded from the study for several reasons, including difference of epidemiological and clinical features of PTSD in childhood, authors' inexperience in child and adolescent psychiatry, parents' refusal.

### Prevalence of psychiatric disorders

The overall rate of PTSD is relatively high in this sample (36.8%), although consistent with previous studies [7,8]. In accordance with previous studies [9], the results confirm that moderate stress (to which most of the examined subjects were exposed) can induce prolonged PTSD symptoms. These results are consistent with DSM-IV-TR definition of traumatic event that, differently from DSM-III, does not require an outstanding stressor that "would evoke significant symptoms of distress in almost everyone". With respect to the aims of the study, DSM-IV-TR PTSD was the most frequent diagnosis in this population, confirming the 1^st ^hypothesis that PTSD is the psychiatric disorder more strictly associated with trauma. The most frequent comorbid psychiatric disorder was GAD, while major depression was not diagnosed in any subject, although both anxious and depressive symptoms were present in patients affected by current PTSD and GAD, as documented by subjects' self assessment with the Zung scales. The absence of past or current diagnosis of major depression was unforeseen and difficult to explain. However, this result appears reliable. Clinical interviews were conducted independently by four senior psychiatrists with experience in mood disorders. All of them got consistent results. While there are many studies of comorbidity in combat veterans with PTSD, studies of PTSD from other sources of trauma (e.g., disasters, crimes, and civil violence) are just beginning to emerge. Although similarities exist, the comorbidity profiles differ according to the type of trauma experienced and the population studied. Additionally, the evidence suggests that the associated psychiatric disorders are not truly comorbid, but are interwoven with the PTSD [10]. Future studies to assess the psychiatric comorbidity in populations affected by PTSD and exposed to different stressful events are needed to address this issue. A problematic topic is the apparent low life-time prevalence of psychiatric disorders in the examined population. The retrospective nature of these data may account for this result. It is also possible that subjects minimized previous psychiatric symptoms in order to present themselves as fully sane before the explosion for medico-legal reasons, however.

### The proximity to the disaster as risk factor for PTSD

The 2^nd ^hypothesis was partially confirmed. Some (sight of dead or injured people, disruptive consequences on work) but not other (presence at the moment of the explosion, death of relatives in the disaster, personal injuries) indices of more direct exposure to the event were related to the risk of developing PTSD. The proximity to disaster or a more direct involvement in the disaster (e.g., lost of family members, relatives, and property) have been found associated with frequency and intensity of mental symptoms in exposed subjects [11-14].

### Previous stress, familiarity for psychiatric disorders, substance abuse or somatization

The 3^rd ^and 4^th ^hypotheses were not confirmed. History of child mourning or separation, previous trauma or familiarity for psychiatric disorders did not play any major role in the development of PTSD in this population. Furthermore, substance use disorder and somatization disorder were not related with the current diagnosis of PTSD. Also in the study on survivors of the Great Midwestern Floods of 1993 in St. Louis Area [15], virtually no new substance abuse followed the floods, and hence, substance abuse did not develop in response to the disaster or as part of coping with its aftermath. In summary, our findings are inconsistent with causal attribution of civil explosion disaster in the etiology of substance abuse and somatization.

### Trauma related long-lasting psychiatric morbidity

In this population, nearly two years after trauma, most of patients who had suffered PTSD [32/41(78.0%)], still met PTSD criteria. These results confirm the 5^th ^hypothesis that severe stress can cause long-lasting morbidity.

### Implications

The study confirms that PTSD is a frequent and long-lasting consequence of exposure to civil disaster. It suggests that previous stress, familiarity for psychiatric disorders, substance abuse or somatization do not increase the risk of PTSD significantly.

## Abbreviations

CAPS = Clinician Administered Post Traumatic Stress Disorder Scale; GAD = Generalized Anxiety Disorder; PTSD = Post-Traumatic Stress Disorder

## Competing interests

The author(s) declare that they have no competing interest.

## Authors' contributions

MR and AO made the conception and design of the study, contributed to the acquisition of data, and performed the analysis and interpretation of data. MR, AO, AA, BB made the clinical interviews. AA, BB, and NM contributed to the acquisition of data and revision of the manuscript. All authors gave final approval of the final version to be published.
